# Long non-coding RNA metastasis-associated lung adenocarcinoma transcript 1 regulates renal cancer cell migration via cofilin-1

**DOI:** 10.3892/ol.2021.12622

**Published:** 2021-03-08

**Authors:** Yali Zhang, Xinyu Guan, Hao Wang, Yong Wang, Dan Yue, Ruibing Chen

Oncol Lett 20: Article no. 53, 2020; DOI: 10.3892/ol.2020.11914

After the publication of the above article, during a routine examination of the raw data the authors noticed errors in Figs. 4 and S2 in their paper. Essentially, in Fig. 4A, the graph of the migration assay for ACHN-siMALAT1-2+CFL1 (0 h) didn't match with the original pictures: An image from a different experiment had been erroneously selected during the process of assembling the figure. Regarding Fig. S2, the blot of the internal standard GAPDH didn't match with the raw data.

The corrected versions of Figs. S2 and 4 are shown below and opposite, respectively. Note that the revisions made to these figures do not affect the overall conclusions reported in the paper. The authors are grateful to the Editor of *Oncology Letters* for allowing them the opportunity to publish this Corrigendum, and apologize to the readership for any inconvenience caused.

## Figures and Tables

**Figure S2.**
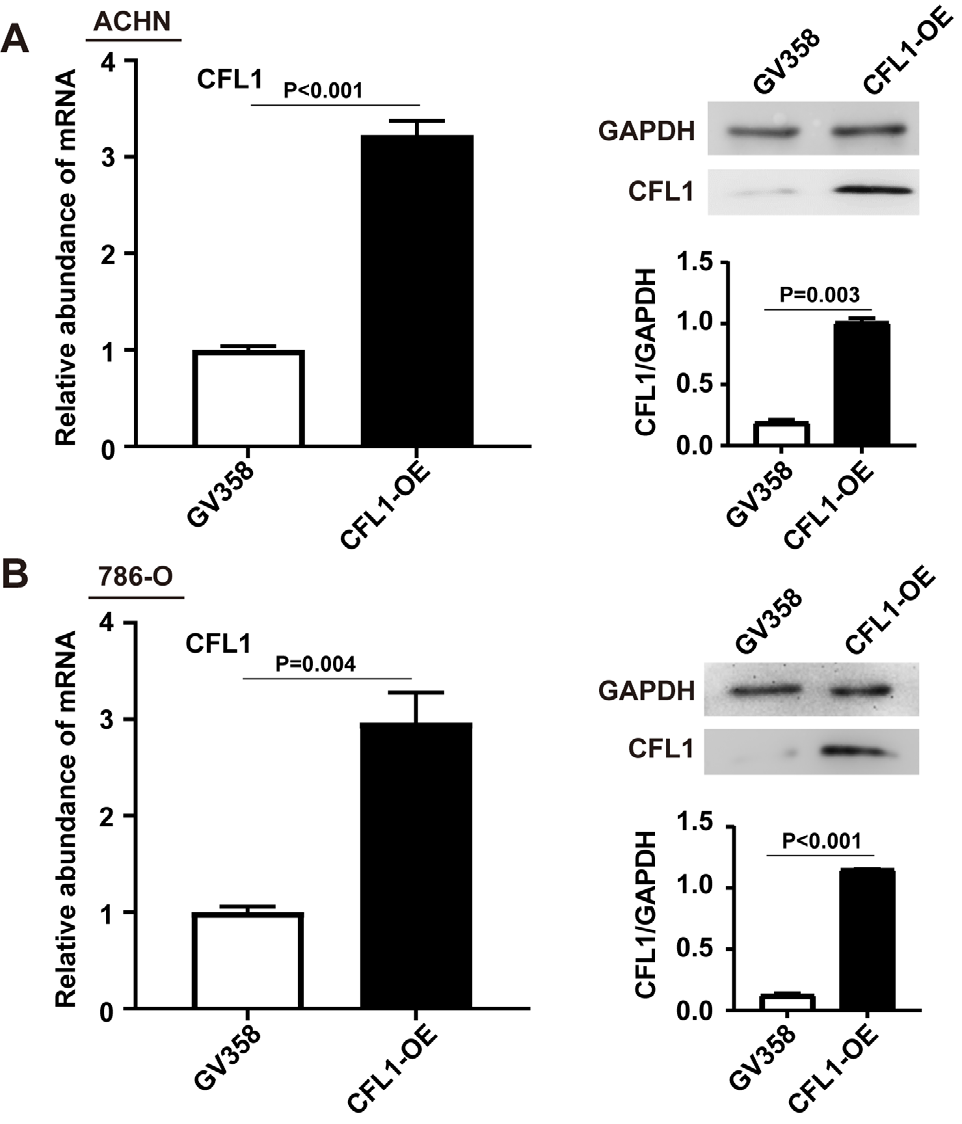
RT-qPCR and western blotting analyses of CFL1 in ACHN (A) and 786-O (B) cells transfected with CFL1 overexpression plasmids and the corresponding control cells transfected with the empty vector. The data are shown as mean ± SD (n=3) and unpaired Student's t-test was used for statistical analysis. CFL1, cofilin-1; OE, overexpression.

**Figure 4.**
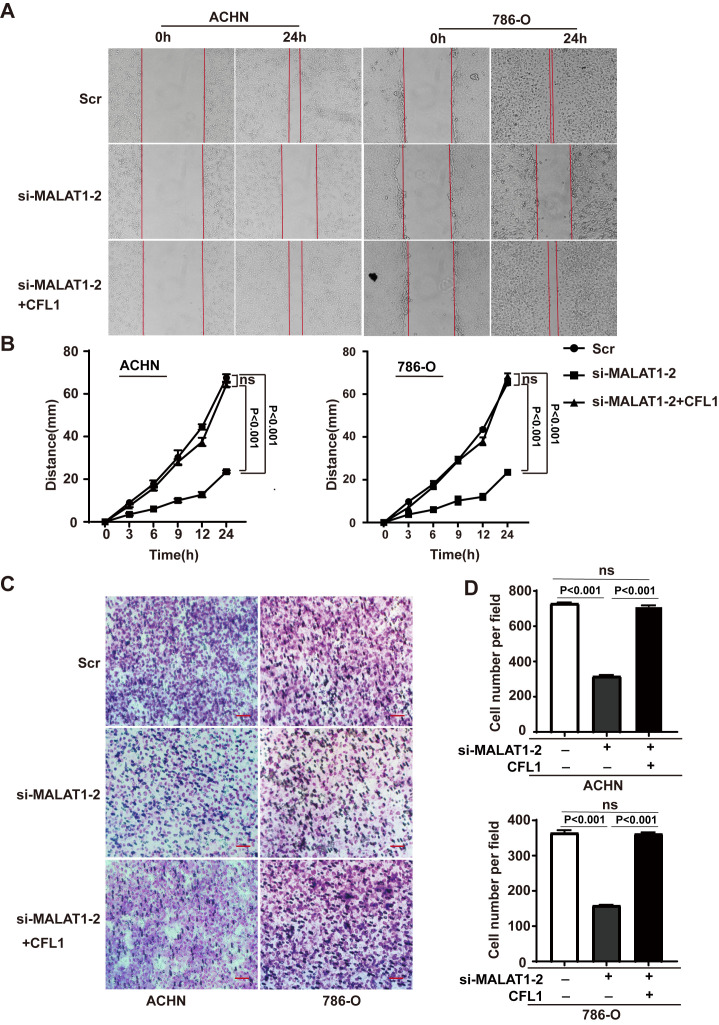
MALAT1 regulates the migration and invasion of renal cell carcinoma cells through CFL1. (A) Cell migration assay of siMALAT1 cells and cells transfected with both siMALAT1 and CFL1 vector. Left panel, ACHN; right panel, 786-O. Representative images of the cells at 0 and 24 h are shown. Magnification, ×100. (B) Quantification of cell migration assay. Migration distances are shown as the mean ± SD of three independent analyses, and one-way ANOVA followed by Tukey's post hoc test was used for statistical analysis. Left panel, ACHN; right panel, 786-O. (C) Invasion assay of the kidney renal clear cell carcinoma cells with indicated treatments. Left panel, ACHN; right panel, 786-O. Representative images are shown. Magnification, ×200. Scale bar, 100 µm. (D) Quantification of the invasion assay. Results are shown as the mean ± SD of three independent analyses and one-way ANOVA followed by Tukey's post hoc test was used for statistical analysis. Upper panel, ACHN; lower panel, 786-O. ns, not significant; MALAT1, metastasis-associated lung adenocarcinoma transcript 1; si, small interfering; CFL1, cofilin-1; SD, standard deviation; ANOVA, analysis of variance.

